# Long noncoding RNA *MALAT1* regulates autophagy associated chemoresistance via miR-23b-3p sequestration in gastric cancer

**DOI:** 10.1186/s12943-017-0743-3

**Published:** 2017-11-21

**Authors:** Hu YiRen, Yu YingCong, You Sunwu, Li Keqin, Tong Xiaochun, Chen Senrui, Chen Ende, Lin XiZhou, Chen Yanfan

**Affiliations:** 1 Department of General Surgery, The third Clinical Institute Affiliated to Wenzhou Medical University, Wenzhou People’s Hospital, Wenzhou, Zhejiang China; 2 Department of Gastroenterology, The third Clinical Institute Affiliated to Wenzhou Medical University, Wenzhou People’s Hospital, Wenzhou, Zhejiang China; 3 Institute of Gastroenterology, Zhejiang University(IGZJU), Hangzhou, Zhejiang China; 4 Department of radiology, Wenzhou No.3 Clinical Institute of Wenzhou Medical University, Wenzhou People’s Hospital, No. 57 Canghou Street, Wenzhou, Zhejiang, 325000 China

**Keywords:** lncRNA, *MALAT1*, Gastric cancer, Chemoresistance, Autophagy

## Abstract

**Background:**

Chemoresistance has long been recognized as a major obstacle in cancer therapy. Clarifying the underlying mechanism of chemoresistance would result in novel strategies to improve patient’s response to chemotherapeutics.

**Methods:**

lncRNA expression levels in gastric cancer (GC) cells was detected by quantitative real-time PCR (qPCR). *MALAT1* shRNAs and overexpression vector were transfected into GC cells to down-regulate or up-regulate *MALAT1* expression. In vitro and in vivo assays were performed to investigate the functional role of *MALAT1* in autophagy associated chemoresistance.

**Results:**

We showed that chemoresistant GC cells had higher levels of *MALAT1* and increased autophagy compared with parental cells. Silencing of *MALAT1* inhibited chemo-induced autophagy, whereas *MALAT1* promoted autophagy in gastric cancer cells. Knockdown of *MALAT1* sensitized GC cells to chemotherapeutics. *MALAT1* acts as a competing endogenous RNA for miR-23b-3p and attenuates the inhibitory effect of miR-23b-3p on ATG12, leading to chemo-induced autophagy and chemoresistance in GC cells.

**Conclusions:**

Taken together, our study revealed a novel mechanism of lncRNA-regulated autophagy-related chemoresistance in GC, casting new lights on the understanding of chemoresistance.

**Electronic supplementary material:**

The online version of this article (10.1186/s12943-017-0743-3) contains supplementary material, which is available to authorized users.

## Background

Gastric cancer (GC) is the fifth most frequent cancer and the third common cause of cancer-related death worldwide [1, 2]. Surgical resection remains the only curative treatment, but most of the patients that suffer from GC are diagnosed at advanced stages [3, 4]. Chemotherapy is the first-line treatment for these patients. However, despite the advancements in the development of novel chemotherapeutic drugs, chemotherapy has only modest efficacy in patients with advanced/metastatic GC. Chemoresistance has long been recognized as a major obstacle in cancer therapy. Thus, the identification of novel molecular mechanisms underlying chemoresistance may improve clinical outcome.

Previous studies have revealed that both intrinsic and acquired chemoresistance come from the genetic and epigenetic modifications occurring in cancer cells [5]. Although the factors responsible for chemoresistance can be diversified, it is well recognized that a cell's capability to manage stress plays a vital role. Accumulating evidence has demonstrated that autophagy, a favored survival strategy that help cancer cells overcome stressful conditions, may play an important role in chemoresistance [6].

Long noncoding RNAs are a class of long (>200 nucleotides) noncoding RNA molecules and have been shown to be a crucial player in cancer biology, including chemoresistance [7–9]. Metastasis-associated lung adenocarcinoma transcript 1 (*MALAT1*) is located on chromosome 11q13 and has been identified to be involved in a wide range of biological and cellular processes, including glycolysis [10], carcinogenesis [11], retinal neurodegeneration [12] and vascular growth [13]. However, how *MALAT1* functions to therapeutically impact GC and the underlying mechanism remains largely unknown.

In the present study, we showed that *MALAT1* is maintained at a higher expression level in chemoresistant GC cells than in chemosensitive cells. Additionally, our data indicated that knockdown of *MALAT1* can sensitize GC cells to chemotherapy by blocking chemotherapy-induced autophagy. Mechanistically, *MALAT1* competitively sequesters miR-23b-3p and relieves the inhibitory effect of miR-23b-3p on ATG12, thereby increasing the expression of ATG12. Thus, our study identified a novel role of *MALAT1* in the regulation of autophagy and suggests that the knockdown of *MALAT1* may sensitize GC cells to chemotherapeutics via suppression of chemotherapy-induced autophagy.

## Methods

### Cell culture

Two human gastric adenocarcinoma cell lines SGC7901 and BGC823 were used in this study. SGC7901/VCR cells were cultured as described previously [14, 15]. SGC7901 and BGC823 were purchased from Cell Bank of the Chinese Academy of Sciences (Shanghai, China). These cell lines were immediately expanded and frozen so that a new aliquot could be thawed every 3 to 4 months from a frozen vial of the same batch of cells. Cells were cultured in Dulbecco’s modified Eagle’s medium (DMEM, Gibco, Carlsbad, CA, USA) supplemented with 10% fetal calf serum, penicillin, and streptomycin (HyClone, Logan, UT, USA) at 37 °C in an atmosphere containing 5% CO_2_.

### RNA preparation and quantitative real-time PCR

Briefly, total RNA was extracted from tissues or cells using TRIzol reagent (Invitrogen, Carlsbad, CA, USA). The quality of total RNA was detected at an A260/A280 ratio using 1% agarose gel electrophoresis. The GoScript Reverse Transcription System (Promega, Madison, Wis) was used to generate complementary DNA. The cDNA template was amplified by real-time RT-PCR using the SYBR Premix Dimmer Eraser kit (TaKaRa, Dalian, China). Gene expression was normalized to GADPH or U6 expression. The real-time PCRs were performed in triplicate and calculated by the 2^−ΔΔCt^ method. Primers used in this study are listed in Additional file 1: Table S1.

### Western blot analysis

Briefly, the total cellular protein was isolated with RIPA cell lysis buffer supplemented with protease inhibitors. Cytosolic protein was isolated using the Mitochondrial and Cytoplasmic Extraction Kit (Thermo Fisher Scientific, Rockford, IL). Protein content was determined by the Bradford assay. Equal amounts (30-50 μg) of proteins were separated by 10% sodium dodecyl sulfate/polyacrylamide gel electrophoresis and transferred to a PVDF Immobilon-P membrane (Millipore, MA). After blocking with 5% skim milk, the membrane was then incubated with indicated primary antibodies and secondary antibodies conjugated to horseradish peroxidase. Antibody-bound proteins were detected by ECL (enhanced chemiluminescence) Western Blotting Substrate (Pierce, Rockford, IL). The band intensity of the western blots and the normalization was analyzed using the ImageJ program (National Institutes of Health, Bethesda, MD). The primary antibodies used include rabbit polyclonal anti-human LC3B (1:500, Abcam), p62 (1:500, Abcam), ATG12 (1:800, Abcam), rabbit monoclonal anti-human caspase-3 (1:500, Abcam), caspase-9 (1:500, Abcam), rabbit monoclonal anti-human cytochrome C (1:500, Epitomics), EZH2 (1:500, Epitomics) and rabbit polyclonal anti-human Actin (1:4,000, Abcam). HRP-conjugated goat anti-rabbit IgG antibody (Abcam) was used as the secondary antibody.

### Electron microscopy

Cells were treated as indicated and fixed with 2.5% glutaraldehyde containing 0.1 mol/L sodium cacodylate. Samples were fixed using 1% osmium tetroxide, followed by dehydration with an increasing concentration gradient of ethanol and propylene oxide. Samples were then embedded, cut into 50-nm sections, and stained with 3% uranyl acetate and lead citrate. Images were acquired using a CM-120 electron microscope (PHILIPS).

### In vitro and in vivo drug-sensitivity assay

For the in vitro drug-sensitivity assay, GC cells were seeded into 96-well plates at a density of 1×10^5^ cells per well. The culture medium containing different concentrations of 5-fluorouracil (5-FU), VCR or cisplatin (CDDP) was added to each well. Forty-eight hours post cultivation, CCK-8 solution (10μl per 100μl of medium in each well) was added to each well and incubated for 2 h. The absorbance was measured by scanning with a microplate reader (MRX; Dynex Technologies, West Sussex, United Kingdom) at 450 nm. Each group comprised six replicates, and the experiments were repeated at least 3 times. Then, the IC50 values for each drug were calculated.

All animal experiments were performed in the animal laboratory center of Wenzhou No.3 Clinical Institute of Wenzhou Medical University, Wenzhou People's Hospital and in accordance with the Guide for the Care and Use of Laboratory Animals published by the US National Institutes of Health (NIH publication number 85-23, revised 1996) and ARRIVE.

For in vivo experiments, SGC7901/VCR cells (1×10^7^) transfected with the desired vector were subcutaneously injected into the flank area of 4-week-old female athymic nude mice (*n*=4 mice per group). After two weeks, the mice were intraperitoneally injected with CDDP in PBS (10 mg/kg) once every week. Tumor volumes were measured every week with the following formula: 0.5 × length × width^2^ every week. Four weeks post inoculation, the mice were sacrificed and the tumors were photographed.

### Quantification of *MALAT1* and miR-23b-3p expression levels

The copy number of *MALAT1* and miR-23b-3p transcripts per cell was quantified by using a quantitative real-time RT–PCR assay as we described previously [9].

### Plasmid construction, lentiviral construction, and cell transfections

Detailed descriptions of plasmid construction, lentiviral vector construction, and cell transfections can be found in Additional file 2.

### Luciferase reporter assay

The fragments of *MALAT1* containing the putative hsa-miR-23b-3p binding site were chemically synthesized. The corresponding mutants were created by mutating the hsa-miR-23b-3p seed region binding site. Cotransfection of psicheck2, psicheck2-*MALAT1* wt or psicheck2- *MALAT1*-mut (miR-23b-3p) with miR-23b-3p mimics, inhibitors or miRNA NC into GC cells was completed with Lipofectamine-mediated gene transfer. Forty-eight hours after transfection, using 100μl of passive buffer, cells were collected to detect the Renilla luciferase activity with the Dual-Luciferase Reporter Assay System (Promega) in TD-20/20. Forty-eight hours post transfection, the relative luciferase activity was determined after normalizing to Renilla luciferase activity.

### Cytosolic/nuclear fractionation isolation and biotin pull-down assay

Detailed descriptions of the cytosolic/nuclear fractionation isolation and Biotin pull-down assays can be found in Additional file 2.

### Patients and clinical samples

Written consent was obtained from all patients in this study. The human tissue specimens used in this study were approved by and under the censorship of the local ethics committee at Wenzhou No.3 Clinical Institute of Wenzhou Medical University, Wenzhou People's Hospital. Six GC patients, who received neoadjuvant chemotherapy before surgery between 2014 and 2015, were identified from Wenzhou People's Hospital, including 3 chemosensitive and 3 chemoresistant cases. The human specimens were subject to immunohistochemical staining of ATG12 and in situ hybridization of *MALAT1*.

### Statistical analysis

All statistical analyses were performed utilizing SPSS version 17.0 software (Chicago, IL, USA). All data are presented as the mean ± standard deviation from three independent repeats. Unless otherwise noted, the differences between two groups were analyzed using Student's *t*-test.

## Results

### Chemoresistant GC cells demonstrate blunted chemosensitivity compared to parental cells

**Fig. 1:**
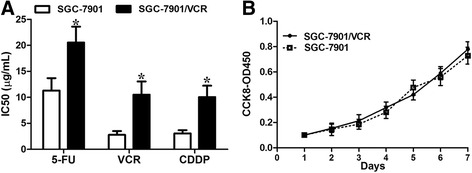
SGC7901/VCR had increased chemoresistance. **a** SGC7901/VCR cells harbored increased IC50 values compared with SGC7901 cells in response to chemotherapeutics. **b** The cell growth rates were determined by performing CCK-8 proliferation assays. SGC7901/VCR cells had similar cell proliferation rate, relative to control cells, in the absence of chemotherapeutics. The data are presented as the means ± S.D. of values obtained in 3 independent experiments. *, *p* < 0.05

The established chemoresistant cell lines SGC7901/VCR (vincristine) derived from human GC cell line SGC7901 was obtained from Professor D. Fan [14, 15]. To confirm the chemosensitivity of SGC7901/VCR cells compared to SGC7901 cells, CCK-8 assays were performed to measure changes in cell proliferation and viability. As demonstrated in Fig. 1a, SGC7901/VCR cells had an enhanced resistance to cisplatin compared to SGC7901 cells. It is worth noting that SGC7901 and SGC7901/VCR demonstrated similar proliferation rates in the absence of chemotherapeutics (Fig. 1b)

.

### Chemoresistant GC cells exhibit increased autophagy

**Fig. 2:**
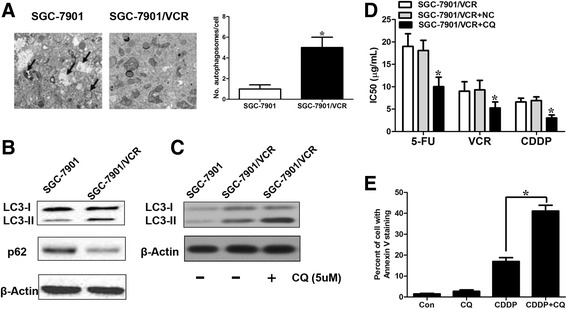
SGC7901/VCR cells exhibit increased autophagy. **a** Autophagy was evaluated in SGC7901/VCR cells that exhibited chemoresistance using transmission electron microscopy. The data were quantified by counting the number of autophagosomes per cross-sectioned cell. **b** Autophagosome formation in whole cell lysates was determined by Western blot analysis using LC3 and p62 antibodies. The top band (16 kilodaltons) represents LC3-I, and the bottom band (14 kilodaltons) represents LC3-II. **c** SGC7901/VCR cells and their parental cell lines were treated with 10 mmol/L CQ for 24 hours before being subjected to Western blot analysis for LC3 expression. **d** CQ greatly enhanced the sensitivity of SGC7901/VCR cells to chemotherapeutic agents. **e** flow cytometric analysis of Annexin V staining. The data are presented as the means ± S.D. of values obtained in 3 independent experiments. *, *p* < 0.05

Autophagy, a conserved lysosome-mediated intracellular degradation system, could protect cancer cells during stress conditions [16]. Autophagy can be induced by chemotherapeutics, and this process relieves cancer cells from chemotherapy-associated cellular damage, thereby contributing to chemoresistance [17]. Thus, we hypothesized that chemoresistant GC cells may have increased autophagy. We utilized transmission electron microscopy (TEM) and western blot analysis to evaluate autophagosomes. TEM demonstrated a substantial increase in the accumulation of autophagic vesicles in SGC7901/VCR cells compared with parental cells (Fig. 2a). LC3 was used as a measure of autophagy activation and the conversion of LC3-I to LC3-II is regarded as a hallmark of autophagy. P62, an adaptor protein that interacts with LC3-II , is also considered as a hallmark of autophagy [18]. As expected, SGC7901/VCR cells expressed a higher LC3-II to LC3-I ratio and a decreased expression level of p62 compared with SGC7901 cells (Fig. 2b). SGC7901/VCR cells were treated with chloroquine (CQ), an autophagy-lysosomal inhibitor, and a further elevation in the LC3-II level was observed, indicating an increase in the autophagic flux of chemoresistant cells (Fig. 2c). Next, we sought to explore whether chemotherapy-induced autophagy affects chemotherapeutic efficacy. We treated SGC7901/VCR cells with CQ and found that co-treatment of SGC7901/VCR with CQ (5 μM) and cisplatin caused greater cytotoxicity than treatment with cisplatin alone as evidenced by the decreased IC50 concentration (Fig. 2d) and increased apoptosis rates (Fig. 2e and Additional file 1: Figure S1).

### *MALAT1* promotes autophagy-associated chemoresistance in GC cells

**Fig. 3:**
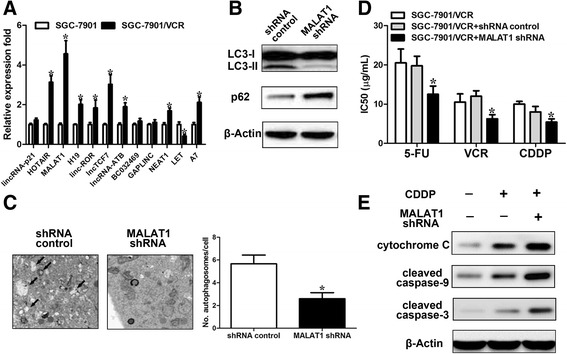
*MALAT1* promotes autophagy in GC cells. **a** Relative mRNA levels of specific lncRNAs in SGC7901/VCR and SGC7901 cells using real-time PCR. **b** SGC7901/VCR cells stably transfected with shRNA-*MALAT1* or a control were subjected to Western blot analysis of LC3-II and p62. **c** Autophagy was evaluated using transmission electron microscopy in SGC7901/VCR stably transfected with shRNA-*MALAT1* or a control. **d** Silencing of *MALAT1* sensitized SGC7901/VCR cells to chemotherapeutic agents as evidenced by decreased IC50 values. **e** SGC7901/VCR cells were treated with the cisplatin (10 μg/ml) for 24 h. Total protein as well as cytoplasmic protein fractions were isolated, and the indicated proteins were detected by Western blot. The data are presented as the means ± S.D. of values obtained in 3 independent experiments. *, *p* < 0.05

We hypothesized that lncRNAs may play a role in the regulation of autophagy. We first determined the expression of several well-known lncRNAs (*lincRNA-p21*, *HOTAIR*, *MALAT1*, *H19*, *linc-ROR*, *lncTCF7*, *lncRNA-ATB*, *BC032469*, *LET*, *GAPLINC*, *NEAT1*, and *A7*) in the SGC7901 and SGC7901/VCR cell lines using real-time quantitative real-time polymerase chain reaction (qRT-PCR) analysis. Of these lncRNAs, *MALAT1* was expressed at higher levels in SGC7901/VCR cells than in SGC7901 cells (Fig. 3a). *MALAT1* is upregulated in a wide range of types of cancer, including GC, according to the Cancer Genome Atlas (TCGA) database (Additional file 1: Figure S2). Similar results were obtained from data in the Gene Expression Omnibus (GEO) database (GES50710, GSE47850, GSE58828), showing that the expression level of *MALAT1* was higher in GC tissues than that in adjacent normal tissues. As *MALAT1* was frequently upregulated in GC and can be induced by chemotherapy, we wanted to explore whether the upregulation of *MALTA1* might play a role in the regulation of chemotherapeutic efficacy via promoting autophagy. We constructed *MALAT1* stably knocked-down cells with an shRNA sequence targeting *MALAT1* that has been shown to be efficient in a number of studies [19, 20]. We found that knockdown of *MALAT1* (Additional file 1: Figure S3a) greatly inhibited autophagy, as indicated by the attenuated LC3-II to LC3-I ratio, p62 protein level (Fig. 3b) and LC3 dots (Fig. 3c). In the functional aspect, we found that knockdown of *MALAT1* sensitized SGC7901/VCR cells to cisplatin as illustrated by decreased IC50 concentration (Fig. 3d) and increased expression levels of cleaved caspase-3 and caspase-9 (Fig. 3e). Furthermore, overexpression of *MALAT1* (Additional file 1: Figure S3b) enhanced autophagy (Additional file 1: Figure S4a,b). To further consolidate our conclusions, we examined the effect of *MALAT1* on autophagy in the GC cell line BGC823. Treatment of CDDP (10 μg/ml) for 24 h induced a significant upregulation of *MALAT1* (Additional file 1: Figure S4c) and activation of autophagy, while *MALAT1* knockdown blunted the autophagic response to cisplatin (Additional file 1: Figure S4d) compared to cells transfected with a control vector. Ectopic expression of *MALAT1* induced chemoresistance in SGC7901 cells as illustrated by decreased cleaved caspase-3 level in response to cisplatin (Additional file 1: Figure S4e). Knockdown of lncRNA-ATB did not have significant effect on the autophagy of SGC7901/VCR cells (Additional file 1: Figure S4f). These data suggest that knockdown of *MALTA1* inhibits the autophagic response in GC cells.

To explore the mechanism by which *MALAT1* was regulated, firstly, we found high enrichment of H3K27Ac at the promoter region of *MALAT1* with UCSC Genome Bioinformatics (Additional file 1: Figure S5a). Furthermore, we found high enrichment of H3K27Ac in gastric cancer tissues compared with normal tissues at the promoter of *MALAT1* (Additional file 1: Figure S5b). We speculate that histone acetylation activation might contribute to the upregulation of *MALAT1* in gastric cancer tissues. Using the CHIP assay, the high enrichment of H3K27Ac was also found in the SGC7901/VCR cells compared to SGC7901 cells at the promoter of *MALAT1* (Additional file 1: Figure S5c). To further consolidate our hypothesis, we treated SGC7901 cells with the histone deacetylase inhibitor trichostatin A (TSA). We found that *MALAT1* was upregulated by the histone deacetylase inhibitor trichostatin A (TSA) (Additional file 1: Figure S5d). Taken together, histone acetylation is involved in the upregulation of *MALAT1*.

We found that the expression of *MALAT1* were significantly upregulated in chemoresistant patients compared with that in chemosensitive patients (Additional file 1: Figure S6a). We did observe decreased expression of LC3B in chemosensitive patients compared with chemoresistant patients (Additional file 1: Figure S6a). According to data from the KMPlot database, we found that high *MALAT1* expression resulted in a poorer disease-free survival (DFS, *n*=153, *p*=0.049) and overall survival (OS, *n*=153, *p*=0,039) in patients who had received 5-Fu-based adjuvant therapy (Additional file 1: Figure S6b).

### ATG12 is a downstream effector in *MALAT1*-mediated autophagy in GC cells

**Fig. 4:**
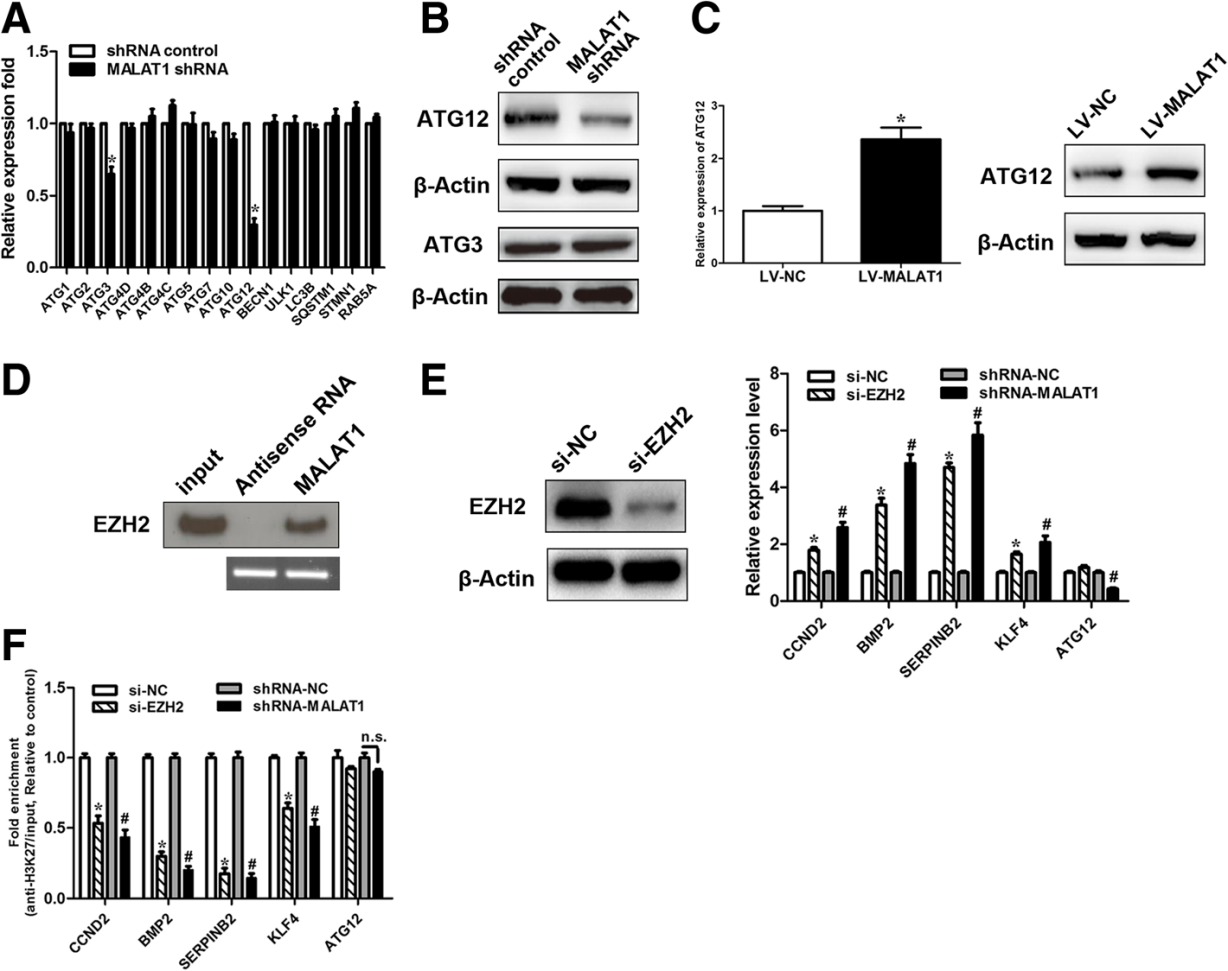
*MALAT1* regulates ATG12. **a** The mRNA expression of the indicated autophagy related genes was measured using real-time PCR in SGC7901/VCR cells stably transfected with shRNA-*MALAT1* or a control. Student *t* tests were used to determine the statistical significance of the differences between the groups. **b** Western blot analysis of ATG12 and ATG3 was performed in SGC7901/VCR cells stably transfected with shRNA-*MALAT1* or a control. **c** The mRNA or protein levels of ATG12 were determined using real-time PCR and Western blot analysis in SGC7901/VCR cells stably transfected with shRNA-*MALAT1* or a control. **d** Biotinylated *MALAT1* or antisense RNA was incubated with cell extracts of SGC7901/VCR cells, targeted with streptavidin beads, and washed, and the associated proteins were resolved on a gel. Western blot analysis detected the specific association of EZH2 and *MALAT1* (n=3). **e** EZH2 knockdown efficiency was confirmed by Western blot. qRT-PCR analysis of putative PRC2 target genes after *MALAT1* and EZH2 knockdown, respectively. **f** ChIP analysis of H3K27 trimethylation status of candidate EZH2 target genes after knockdown assay. The data are presented as the means ± S.D. of values obtained in 3 independent experiments. #, *p* < 0.05. *, *p* < 0.05. n.s., not significant

Next, we sought to identify the underlying mechanism of *MALAT1*-mediated autophagy. First, using qRT-PCR, we determined the effect of *MALAT1* on the expression of the identified regulators of autophagy ATG1, ATG2, ATG3, ATG4D, ATG4B, ATG4C, ATG5, ATG7, ATG12, ATG13, ATG14, BECN1, ULK1, LC3B, SQSTM1, STMN1, and RAB5A with qRT-PCR analysis. We demonstrated that *MALAT1* silencing greatly reduced the mRNA level of ATG12 (Fig. 4a). Furthermore, western blot analysis illustrated that the suppression of *MALAT1* substantially downregulated the protein level of ATG12; yet, its effect on ATG3 was not significant (Fig. 4b). Overexpression of *MALAT1* increased both ATG12 mRNA and protein expression levels (Fig. 4c). As lncRNAs have been shown to be involved in multiple levels of genes regulation, including transcriptional regulation via recruitment of chromatin-modifying complexes, and post-transcriptional regulation by interactions with mRNAs, miRNAs, and proteins [7]. Previous studies have revealed that *MALAT1* may exert biological effects via interacting with chromatin-modifying complexes, such as EZH2 [21, 22], by acting as competitive endogenous RNAs (ceRNAs) [22], or by affecting protein stability [10] or protein phosphorylation status [12]. As anticipated, RNA pull-down experiments revealed that *MALAT1* specifically interacted with EZH2 (Fig. 4d). We then explored whether *MALAT1* or EZH2 silencing had any effect on the transcript levels of polycomb repressive complex 2 (PRC2) targets. qRT-PCR analysis revealed that either *MALAT1* or EZH2 silencing suppressed the mRNA levels of previously identified PRC2 targets [23], including CCDN2, BMP2, KLF4 and SERPINB2. However, knockdown of EZH2 had no significant effect on the transcript levels of ATG12, despite that a similar depression of these target genes being observed in EZH2-downregulated cells (Fig. 4e). Furthermore, knockdown of EZH2 resulted in decreased trimethylation level of H3K27 in the promoter regions of CCND2, BMP2 and KLF4 by ChIP-qPCR analysis. However, no significant change in the trimethylation level of H3K27 in the promoter region of ATG12 was observed (Fig. 4f). These data suggest that the effect of *MALAT1* on ATG12 may not dependent on PRC2. Because *MALAT1* regulates ATG12 transcriptionally, we hypothesized that *MALAT1* may act as a miRNA sequestrant for regulating ATG12 expression.

**Fig. 5:**
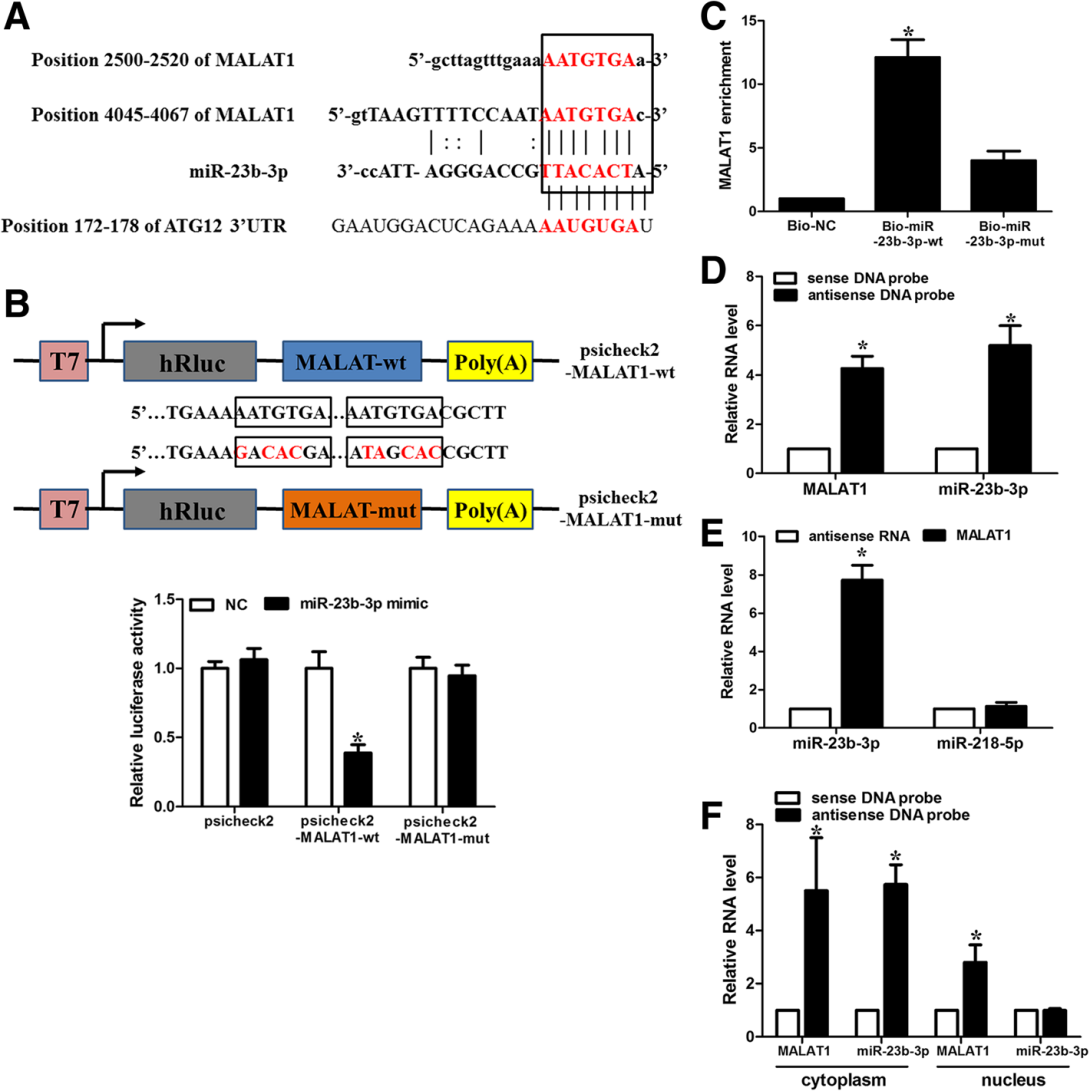
*MALAT1* is a molecular sponge for miR-23b-3p. **a** Illustration of the base pairing between miR-23b-3p and *MALAT1*. The base pairing between miR-23b-3p and ATG12 3’UTR is also shown. **b** Schematic representation of psicheck2-based luciferase reporter plasmid containing wild-type *MALAT1* (psicheck2-*MALAT1*-wt) and a mutant reporter construct in which two putative miR-23b-3p binding sites were mutated (psicheck2-*MALAT1*-mut), and mutated bases are indicated in red. miR-23b-3p or control mimics were transfected into SGC7901/VCR cells together with the indicated psicheck2-based luciferase reporter construct. Twenty-four hours after transfection, reporter activity was measured and plotted after normalizing with respect to Renilla luciferase activity. **c** miR-23b-3p can bind directly to *MALAT1*. SGC7901/VCR cells were transfected with biotinylated wild-type miR-23b-3p (Bio-23b-3p-wt) or biotinylated mutant miR-23b-3p (Bio-23b-3p-mut). A biotinylated miRNA that is not complementary to *MALAT1* was used as a negative control (Bio-NC). Forty-eight hours after transfection, cells were harvested for biotin-based pull-down assay. *MALAT1* expression levels were analysed by real-time PCR. *, *p*<0.05 versus Bio-NC. **d** Lysates from SGC7901/VCR cells were incubated with in vitro-synthesized biotin-labeled sense or antisense DNA probes against *MALAT1* for biotin pull-down assay, followed by real-time RT–PCR analysis to examine miR-23b-3p levels. **e** Lysates from SGC7901/VCR cells were incubated with in vitro-synthesized biotin-labeled *MALAT1* and antisense RNA for biotin pull-down assay, followed by real-time RT–PCR analysis to examine miR-23b-3p and miR-218-5p levels. **f** SGC7901/VCR cells were subjected to cytoplasm or nucleus fractionation before each fraction was incubated with in vitro-synthesized biotin-labeled sense or antisense DNA probes of *MALAT1* for biotin pull-down assay, followed by real-time RT–PCR analysis to examine miR-23b-3p levels. Data shown are means ± S.D. (*n* = 3; *, *p* < 0.05, two-tailed *t*-test). *, *p* < 0.05

Bioinformatics analysis with starbase v2.0 revealed that *MALAT1* formed complementary base pairing (two putative 7-mer complementary sequences) with miR-23b-3p (Fig. 5a), which has been shown to target ATG12 and regulates autophagy-associated chemoresistance in gastric cancer in the previous study [24]. The expression level of *MALAT1* was about one half of miR-23b-3p in SGC7901/VCR cells (Additional file 1: Figure S7a). First, we examined whether *MALAT1* was capable of interacting with miR-23b-3p. To confirm the physical interaction between *MALAT1* and miR-23b-3p, we constructed luciferase reporter constructs (psicheck2). The luciferase reporter constructs were cotransfected with miR-23b-3p mimics or inhibitors into GC cells. As illustrated in Fig. 5b, miR-23b-3p mimics reduced the luciferase activity of the construct containing wild-type (WT) *MALAT1*. However, luciferase activity of constructs containing mutant *MALAT1* was comparable to that of control cells. Our data indicate a direct interaction between *MALAT1* and miR-23b-3p. We employed the biotin–avidin pull-down assay to determine whether miR-23b-3p could pull down *MALAT1*. SGC-7901/VCR cells transfected with biotinylated miR-23b-3p were collected for the biotin–streptavidin pull-down assay. *MALAT1* was pulled down and analyzed by qRT-PCR. As anticipated, miR-23b-3p successfully pulled down *MALAT1*; however, mutations in the binding site between *MALAT1* and miR-23b-3p disturbed of the pull down of *MALAT1* by miR-23b-3p (Fig. 5c). We also used in vitro-synthesized biotinylated *MALAT1* probe and biotinylated antisense DNA probe-enriched endogenous *MALAT1* to pull down miR-23b-3p. MiR-218-5p, which formed no base pairing with *MALAT1*, was used as a negative control. We showed that *MALAT1* specifically pulled down miR-23b-3p (Fig. 5d and e), however, *MALAT1* was not able to pull down miR-218-5p (Fig. 5e). These data confirmed that *MALAT1* physically interacts with miR-23b-3p in a sequence-specific manner. Next, we chose to test where the interaction of *MALAT1* with miR-23b-3p occurs. The results revealed that miR-23b-3p was mainly expressed in the cytoplasm, while *MALAT1* was located in both the nuclear and cytoplasmic fractions of GC cells (Additional file 1: Figure S7b). The antisense DNA probe enriched endogenous *MALAT1* pull down of miR-23b-3p from the cytosolic but not the nuclear fraction (Fig. 5f). Furthermore, treatment of CDDP (10 μg/ml) for 24 h induced a downregulation of miR-23b-3p in GC cells (Additional file 1: Figure S8a).

**Fig. 6:**
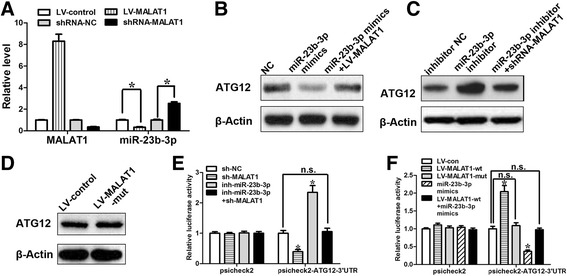
*MALAT1* relieves the inhibitory effect of miR-23b-3p on ATG12. **a** SGC7901/VCR cells were infected with lentiviruses expressing control shRNA or *MALAT1* shRNA. Forty-eight hours after infection, total RNA was subjected to real-time RT–PCR analysis. **b** The protein levels of ATG12 in SGC7901/VCR cells transfected with mimic control, miR-23b-3p mimics, miR-23b-3p mimics +LV-*MALAT1*. **c** The protein levels of ATG12 in SGC7901/VCR cells transfected with inhibitor control, miR-23b-3p inhibitor, miR-23b-3p inhibitor+shRNA-*MALAT1*. **d** Overexpression of miR-23b-3p binding-defective *MALAT1* had no significant effect on the expression of ATG12 in SGC7901/VCR cells. **e**, **f** Luciferase activity in SGC7901/VCR cells transfected with luciferase reporters containing ATG12 3’-UTR or nothing. Data are represented as the relative ratio of firefly luciferase activity to Renilla luciferase activity. Error bars represent the mean±S.D. of triplicate experiments. *, *p* < 0.05

In the previous study, An et al [24] demonstrated that ATG12 is a *bona fide* target of miR-23b-3p and that its expression can be regulated by miR-23b-3p in GC. Finally, given that miR-23b-3p was capable of targeting both *MALAT1* and ATG12, we tested whether *MALAT1* could competitively sequester miR-23b-3p and relieves the inhibitory effect of miR-23b-3p on ATG12. qRT-PCR analysis revealed that *MALAT1* silencing increased, whereas ectopic expression of *MALAT1* increased the levels of miR-23b-3p (Fig. 6a). miR-23b-3p had no effect on the expression level of *MALAT1* (Additional file 1: Figure S8b). As *MALAT1* shares regulatory miR-23b-3p with ATG12, we wanted to determine if *MALAT1* regulates ATG12 through its regulatory role on miR-23b-3p. Western blot analysis showed that *MALAT1* overexpression attenuated the decrease in the protein expression levels of ATG12 induced by miR-23b-3p mimics (Fig. 6b). In addition, the miR-23b-3p inhibitor-mediated upregulation of ATG12 was greatly reversed by *MALAT1* knockdown (Fig. 6c). Furthermore, overexpression of miR-23b-3p binding-defective *MALAT1* had no significant effect on the expression of ATG12 (Fig. 6d). To reinforce the conclusion, we constructed a luciferase reporter containing 3’-UTR of ATG12. The reporter activity was obviously suppressed by *MALAT1* silencing, while the miR-23b-3p inhibitor relieved this decrease (Fig. 6e). In contrast, the overexpression of WT *MALAT1,* but not the miR-23b-3p binding-defective *MALAT1*, increased the reporter activity, while miR-23b-3p mimics attenuated this increase (Fig. 6f). Taken together, these results suggest that *MALAT1* competitively sequesters miR-23b-3p and attenuates the inhibitory effect of miR-23b-3p on ATG12, thereby elevating the expression of ATG12. To explore the possibility that *MALAT1* might function through modulating other miR-23b-3p target genes, we examined the effect of *MALAT1* on HMGB2, which has been shown to regulate chemoresistance-associated autophagy [24]. We found that *MALAT1* silencing suppressed the expression of ATG12 and HMGB2, whereas co-transfection of miRNA-23b-5p inhibitor attenuated this inhibition (Additional file 1: Figure S8c). The incomplete rescue of HMGB2 by miRNA-23b-3p inhibitor suggests that other mechanisms might also be involved in its regulation.

### *MALAT1* promotes autophagy associated chemoresistance of GC cells via miR-23b-3p

**Fig. 7:**
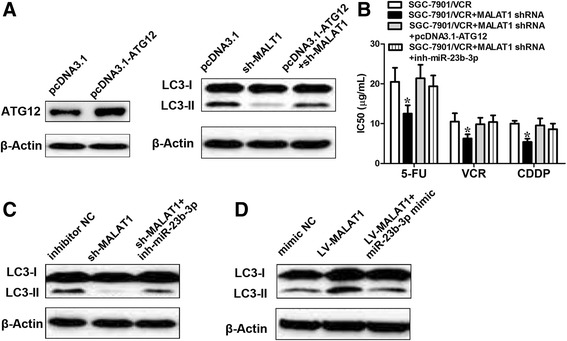
*MALAT1* regulates autophagy via ATG12. **a** Western blot analysis to confirm the efficacy of overexpression of ATG12. SGC7901/VCR cells were transfected with pcDNA3.1- empty vector, shRNA-*MALAT1*, shRNA-*MALAT1*+ pcDNA3.1-ATG12. LC3-II expression was evaluated by Western blot. **b** The decreased IC50 values induced by *MALAT1* knockdown could be relieved by ATG12 or miR-23b-3p inhibitors. **c** SGC7901/VCR cells were transfected with inhibitor NC, shRNA-*MALAT1*, shRNA-*MALAT1*+ miR-23b-3p inhibitor. LC3-II expression was evaluated by Western blot. **d** SGC7901/VCR cells were transfected with inhibitor NC, LV-*MALAT1*, LV-*MALAT1*+ miR-23b-3p mimics. LC3-II expression was evaluated by Western blot. Data shown are means ± S.D. (*n* = 3; two-tailed *t*-test). *, *p* < 0.05

Next, we sought to identify whether *MALAT1* associated chemoresistance was dependent on ATG12 upregulation. In the previous study, ATG12 silencing obviously increased chemosensitivity in GC cells and miR-23b-3p chemosensitizes GC cells by regulating ATG12 [24]. In the present study, we showed that ectopic expression of ATG12 alleviated the suppressive effect on autophagy induced by *MALAT1* silencing (Fig. 7a). Additionally, we demonstrated that the chemosensitization induced by *MALAT1* suppression could be ameliorated by ATG12 overexpression (Fig. 7b). We also found that miR-23b-3p inhibitors relieved the inhibition of autophagy caused by *MALAT1* suppression (Fig. 7c). Similarly, miR-23b-3p inhibitors abrogated the chemosensitization induced by *MALAT1* knockdown (Fig. 7b). Furthermore, the activation of autophagic response induced by *MALAT1* overexpression could be ameliorated by miR-23b-3p inhibitors (Fig. 7d). These data suggest that *MALAT1* promotes autophagy-associated chemoresistance of GC cells via sequestration of miR-23b-3p.

**Fig. 8:**
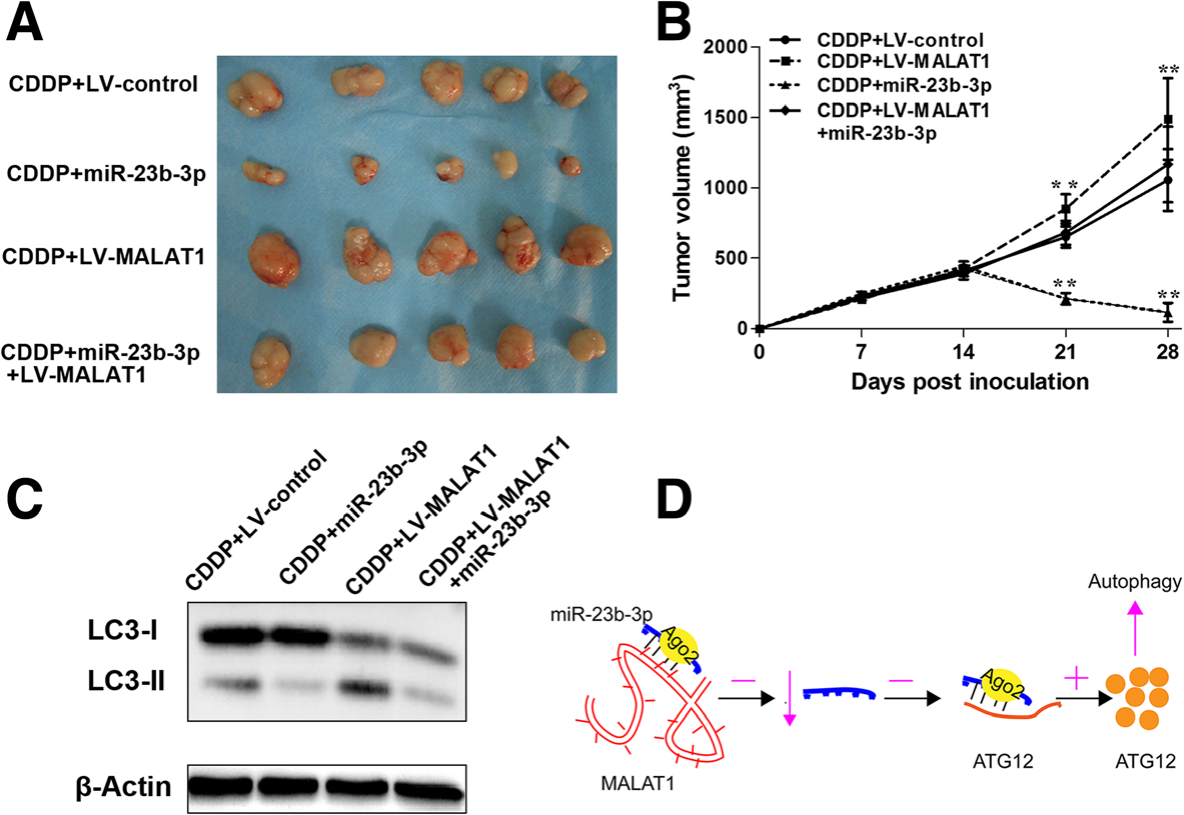
The restoration of miR-23b-3p reversed the drug resistance induced by *MALAT1* overexpression in vivo. 1.0 × 10^7^ SGC7901/VCR cells stably transfected with lenti-*MALAT1* or lenti-NC or lenti-*MALAT1*+miR-23b-3p were subcutaneously injected into the flank of nude mice. Two weeks later, the mice were intraperitoneally injected with PBS containing CDDP (10 mg/kg) once per week. The mice were humanely killed on day 28, and the tumors were measured and photographed. Tumor volumes (**a**) and tumor growth curves (**b**) of subcutaneous implantation models of GC are shown. **c** Total protein fractions were isolated from cells derived from 3 representative xenograft samples from each group, and the LC3 proteins were detected by Western blot analysis. Data shown are means ± S.D. (*n* = 3; **, *p* < 0.01, two-tailed *t*-test). **d** A summary diagram presenting the interaction between *MALAT1*, miR-23b-3p, ATG12 and their effect on autophagy-associatated chemoresistance

We employed the xenograft model to confirm the effects of *MALAT1* and miR-23b-3p on chemosensitivity. Consistent with *in vitro* observations, we observed that while chemotherapeutics greatly decreased tumor volume, *MALAT1* overexpression significantly increased the chemoresistance (Fig. 8a, b). The drug resistance induced by *MALAT1* overexpression could be reversed by ectopic miR-23b-3p expression (Fig. 8a, b). Furthermore, western blot analysis revealed that the tumor LC3-II to LC3-I ration from the miR-23b-3p overexpression group was lower, which was rescued by overexpression of *MALAT1* (Fig. 8c). A summary diagram presenting the interaction between *MALAT1*, miR-23b-3p, ATG12 and their effect on autophagy-associatated chemoresistance is shown in Fig. 8d. Furthermore, we found that the expression of miR-23b-3p was downregulated in chemoresistant (Additional file 1: Figure S9a) patients, whereas the expression of ATG12 was increased in chemoresistant patients. According to data from The KMPlot database (TCGA), patients with low miR-23b-3p expression (Additional file 1: Figure S9b) and high ATG12 expression (Additional file 1: Figure S9c) had a decreased DFS and OS in patients who received 5-Fu-based adjuvant therapy.

## Discussion

Chemotherapy remains the first line of therapy for advanced/metastatic GC. Chemoresistance, whether primary or acquired, is the main obstacle in the majority of cancers.^5^ Although great efforts have been taken into clarifying the molecular mechanisms of the chemoresistance [5], the precise mechanisms remain largely unknown. Autophagy, an evolutionarily conserved, lysosome-mediated intracellular degradation system that is important for cellular homeostasis, development and differentiation [17], has emerged as a new player in chemoresistance [6]. Accumulating evidence has demonstrated that lncRNAs contribute to the chemoresistance in a wide ranges of cancers [7, 25, 26]. In the present study, we bridged the gap between lncRNA and autophagy associated chemoresistance in GC. We showed that *MALAT1* competes with ATG12 mRNA for miR-23b-3p binding and ameliorates the suppressive effect of miR-23b-3p on ATG12.

Previous studies have contradictory opinions about the roles of autophagy in cancer progression. Qu et al [27] showed that disruption of autophagy may promote the tumorigenesis. Autophagy has also been shown to protect cells from stress conditions, such as starvation, chemotherapeutics or radiotherapeutics [6, 17, 24]. Various kinds of stimuli, including anticancer cancer treatment, hypoxia and starvation may induce autophagy [16, 17, 24, 28]. Chemotherapy-induced autophagy have been revealed to help cancer cells escape from deadly cell damage, thereby contributing to chemoresistance [17, 24]. Thus, targeting autophagy-associated regulators may be a potential strategy for eliminating therapeutic resistance in cancer.

LncRNAs have been demonstrated as potent prognostic indicators in various cancers [8, 9, 29]. A growing volume of literature illustrates the association between treatment efficacy (chemotherapy or radiotherapy sensitivity) and tumor lncRNA expression [7, 25, 26]. A few studies that have also established a link between lncRNAs and autophagy-associated chemoresistance [30, 31].

Our study showed that *MALAT1*, a lncRNA overexpressed in a wide range of cancers [11, 29, 32], was upregulated in chemoresistant GC cells. Some studies have revealed that *MALAT1* may be an inducer of autophagy [33–35], few studies have concentrated on the association between *MALAT1* and chemosensitivity. Thus, its role in chemoresistance requires further research. In the present study, we explored the effect of *MALAT1* on chemosensitivity in GC cells. We showed that *MALAT1* competitively sequesters miR-23b-3p and relieves the inhibitory effect of miR-23b-3p on ATG12, thereby increasing the expression of ATG12. ATG12, a vital regulator of autophagy, is upregulated in various cancers [17, 24, 36]. Our data revealed that the chemosensitizing effect of *MALAT1* knockdown was independent of proliferation. However, one study reported that *MALAT1* promotes pancreatic cancer proliferation via the stimulation of autophagy [34]. Previous studies have also showed that CQ-mediated autophagy suppression inhibited cell proliferation in pancreatic cancer cell lines [37, 38]. However, this contradicts our data, as chemoresistant GC cells and the parental cells demonstrated similar proliferation rates in the absence of chemotherapeutics. Additionally, it is worth noting that similar reports were primarily on pancreatic cancer, which have higher levels of basal autophagy than other types of cancer [37], suggesting that autophagy-associated cell proliferation is cancer specific and requires intensive investigation.

Overall, our study identified the role of *MALAT1* in the chemoresistance of GC. *MALAT1* promotes autophagy by sequestering miR-23b-3p and tittering miR-23b-3p off its target ATG12, thus increasing the level of ATG12 and contributing to autophagy-associated chemoresistance. Our report provides novel insights into the molecular mechanisms underlying chemoresistance.

## Additional files

Additional file 1: Figure S1. Flow cytometric analysis of Annexin V staining. **Figure S2** *MALAT1* expression levels vary across different cancer types in the TCGA database. **Figure S3** (a) *MALAT1* expression was detected in SGC7901/VCR cells by qRT-PCR after transduction of lentiviruses encoding *MALAT1* shRNA or a scrambled shRNA. Northern blot analysis of *MALAT1* expression in SGC7901/VCR cells after transduction of lentiviruses encoding *MALAT1* shRNA or a scrambled shRNA. (b) *MALAT1* expression was detected in SGC7901/VCR cells by qRT-PCR after transfection of lentivirus harboring the full-length human *MALAT1* sequence or the empty vector. The data are presented as the means ± S.D. of values obtained in 3 independent experiments. *, *p* < 0.05. **Figure S4** *MALAT1* promotes autophagy. (a) SGC7901/VCR cells stably transfected with full-length human *MALAT1* sequence or the empty vector were subjected to Western blot analysis of LC3-II and p62. (b) Autophagy was evaluated using transmission electron microscopy in SGC7901/VCR stably transfected with full-length human *MALAT1* sequence or the empty vector. (c) In BGC823 cells, treatment of CDDP (10 μg/ml) for 24 h induced a significant upregulation of *MALAT1* as determined with qRT-PCR analysis. (d) In BGC823 cells, treatment of CDDP (10 μg/ml) for 24 h induced a significant activation of autophagy, while *MALAT1* knockdown blunted the autophagic response to cisplatin. (e) SGC7901 cells transfected with full-length human *MALAT1* sequence or the empty vector were treated with the cisplatin (5 μg/ml) for 24 h, caspase-3 protein was detected by Western blot. The data are presented as the means ± S.D. of values obtained in 3 independent experiments. *, *p* < 0.05. (f) SGC7901/VCR cells stably transfected with shRNA-lncRNA-ATB or a control were subjected to Western blot analysis of LC3-II and p62. **Figure S5** (a) UCSC Genome Bioinformatics Site (http://genome.ucsc.edu/) showed high enrichment of H3K27Ac at the promoter of *MALAT1*. (b) ChIP assays detected the H3K27Ac acetylation at promoter of *MALAT1* in gastric cancer tissues. (c) ChIP assays detected the H3K27Ac acetylation at promoter of *MALAT1* in gastric cancer cells.*, *p* < 0.05, **, *p*< 0.01. (d) The expression of the *MALAT1* transcript (mean ± standard deviation) was detected using RT-PCR after cells were stimulated with varying concentrations of the histone deacetylase inhibitor trichostatin A (TSA) for 24 hr. **Figure S6** (a) Compared with chemosensitive patients, LC3B and *MALAT1* were markedly upregulated in chemoresistant patients using immunohistochemical analysis (for LC3B) and *in situ* hybridization analysis (for *MALAT1*). (b) According to data from The KMPlot database (TCGA), high *MALAT1* expression resulted in a poorer disease-free survival (DFS, *n*=153, *p*=0.049) and overall survival (OS, *n*=153, *p*=0,039) in patients who received 5-Fu based adjuvant therapy. The HRs and *p* values were calculated with log-rank tests. **Figure S7** (a) The copy number of *MALAT1* or miR-23b-3p detected SGC7901/VCR cells, using RT-PCR and standard curves of known copy numbers of plasmid-derived reference standard. Error bars show standard deviation. (b) Cellular characterization of *MALAT1* and miR-23b-3p, the levels of nuclear control transcript (U1), cytoplasmic control transcript (Actin mRNA), and *MALAT1* were assessed by qRT-PCR in nuclear and cytoplasmic fractions in SGC7901/VCR cells. Data are presented as a percentage of U1, Actin and *MALAT1* levels and total levels for each were taken to be 100%. The data are presented as the means ± S.D. of values obtained in 3 independent experiments. **Figure S8** (a) In BGC823 cells, treatment of CDDP (10 μg/ml) for 24 h induced a significant downregulation of miR-23b-3p as determined with qRT-PCR analysis. (b) *MALAT1* expression was detected in SGC7901/VCR cells by qRT-PCR after transduction of lentiviruses encoding miR-23b-3p mimic or control mimic. (c) SGC7901/VCR cells were transfected with sh-NC, sh-*MALAT1*, sh-*MALAT1*+miRNA-23b-3p inhibitor and miR-23b-3p inhibitor. qRT-PCR was performed 48 h post transfection. ATG12 and HMGB2 were determined with qRT-PCR analysis. **Figure S9** (a) Compared with chemosensitive patients, ATG12 was markedly upregulated and miR-23b-3p was downregulated in chemoresistant patients using immunohistochemical analysis (for ATG12) and in situ hybridization analysis (for miR-23b-3p). (b,c) According to data from The KMPlot database (TCGA), low miR-23b-3p and high ATG12 expression resulted in a poorer disease-free survival and overall survival in patients who received 5-Fu based adjuvant therapy. The HRs and *p* values were calculated with log-rank tests. **Table S1** Primer sequence used in this study. (DOCX 2642 kb)

Additional file 2: Suppemental materials and methods. (DOCX 16 kb)

## Abbreviations

GC: Gastric cancer
*HOTAIR*: HOX transcript antisense RNA
lncRNA: Long non-coding RNA
*MALAT1*: Metastasis-associated lung adenocarcinoma transcript 1
