# Supplementary material for: Long noncoding RNA MALAT1 regulates autophagy associated chemoresistance via miR-23b-3p sequestration in gastric cancer
Source: Mol Cancer. 2017 Nov 21;16:174. doi: 10.1186/s12943-017-0743-3 (PMC5699172; doi:10.1186/s12943-017-0743-3)
Supplement: Supplementary file 1 — Flow cytometric analysis of Annexin V staining. Figure S2 MALAT1 expression levels vary across different cancer types in the TCGA database. Figure S3 (a) MALAT1 expression was detected in SGC7901/VCR cells by qRT-PCR after transduction of lentiviruses encoding MALAT1 shRNA or a scrambled shRNA. Northern blot analysis of MALAT1 expression in SGC7901/VCR cells after transduction of lentiviruses encoding MALAT1 shRNA or a scrambled shRNA. (b) MALAT1 expression was detected in SGC7901/VCR cells by qRT-PCR after transfection of lentivirus harboring the full-length human MALAT1 sequence or the empty vector. The data are presented as the means ± S.D. of values obtained in 3 independent experiments. *, p < 0.05. Figure S4 MALAT1 promotes autophagy. (a) SGC7901/VCR cells stably transfected with full-length human MALAT1 sequence or the empty vector were subjected to Western blot analysis of LC3-II and p62. (b) Autophagy was evaluated using transmission electron microscopy in SGC7901/VCR stably transfected with full-length human MALAT1 sequence or the empty vector. (c) In BGC823 cells, treatment of CDDP (10 μg/ml) for 24 h induced a significant upregulation of MALAT1 as determined with qRT-PCR analysis. (d) In BGC823 cells, treatment of CDDP (10 μg/ml) for 24 h induced a significant activation of autophagy, while MALAT1 knockdown blunted the autophagic response to cisplatin. (e) SGC7901 cells transfected with full-length human MALAT1 sequence or the empty vector were treated with the cisplatin (5 μg/ml) for 24 h, caspase-3 protein was detected by Western blot. The data are presented as the means ± S.D. of values obtained in 3 independent experiments. *, p < 0.05. (f) SGC7901/VCR cells stably transfected with shRNA-lncRNA-ATB or a control were subjected to Western blot analysis of LC3-II and p62. Figure S5 (a) UCSC Genome Bioinformatics Site (http://genome.ucsc.edu/) showed high enrichment of H3K27Ac at the promoter of MALAT1. (b) ChIP assays detected the H3K27Ac acetylation at [file 12943_2017_743_MOESM1_ESM.docx]

**Additional file 1**

**
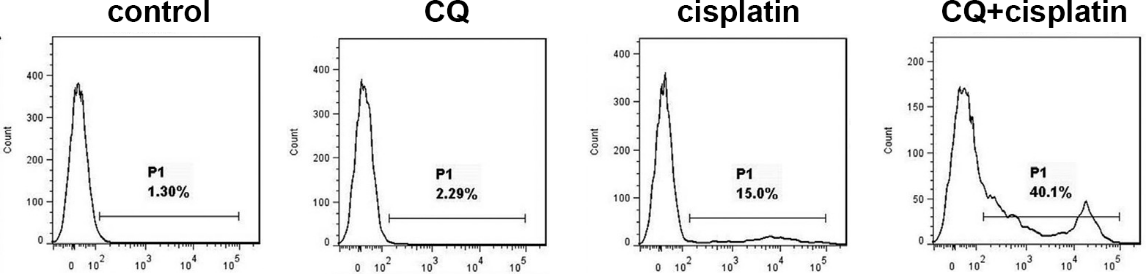
**

**Figure S1** Flow cytometric analysis of Annexin V staining.

**
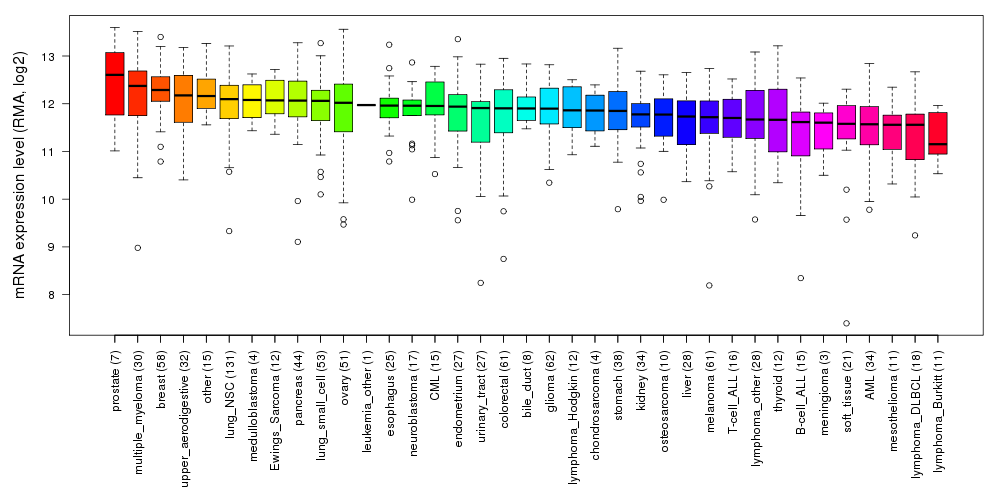
**

**Figure S2** *MALAT1* expression levels vary across different cancer types in the TCGA database.

**
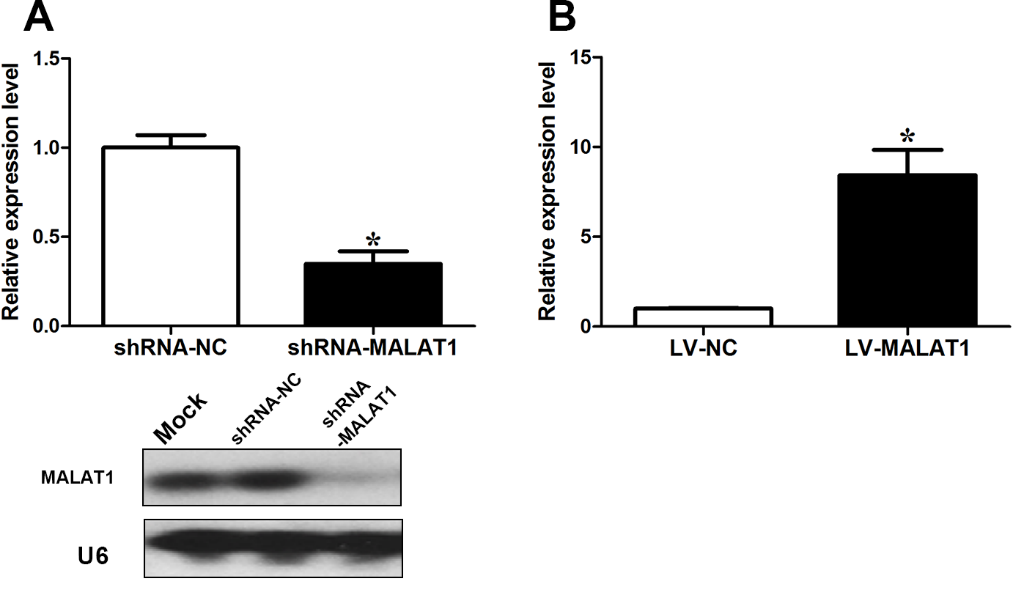
**

**Figure S3** (a) MALAT1 expression was detected in SGC7901/VCR cells by qRT-PCR after transduction of lentiviruses encoding MALAT1 shRNA or a scrambled shRNA. Northern blot analysis of MALAT1 expression in SGC7901/VCR cells after transduction of lentiviruses encoding MALAT1 shRNA or a scrambled shRNA. (b) MALAT1 expression was detected in SGC7901/VCR cells by qRT-PCR after transfection of lentivirus harboring the full-length human MALAT1 sequence or the empty vector. The data are presented as the means ± S.D. of values obtained in 3 independent experiments. *, p < 0.05.


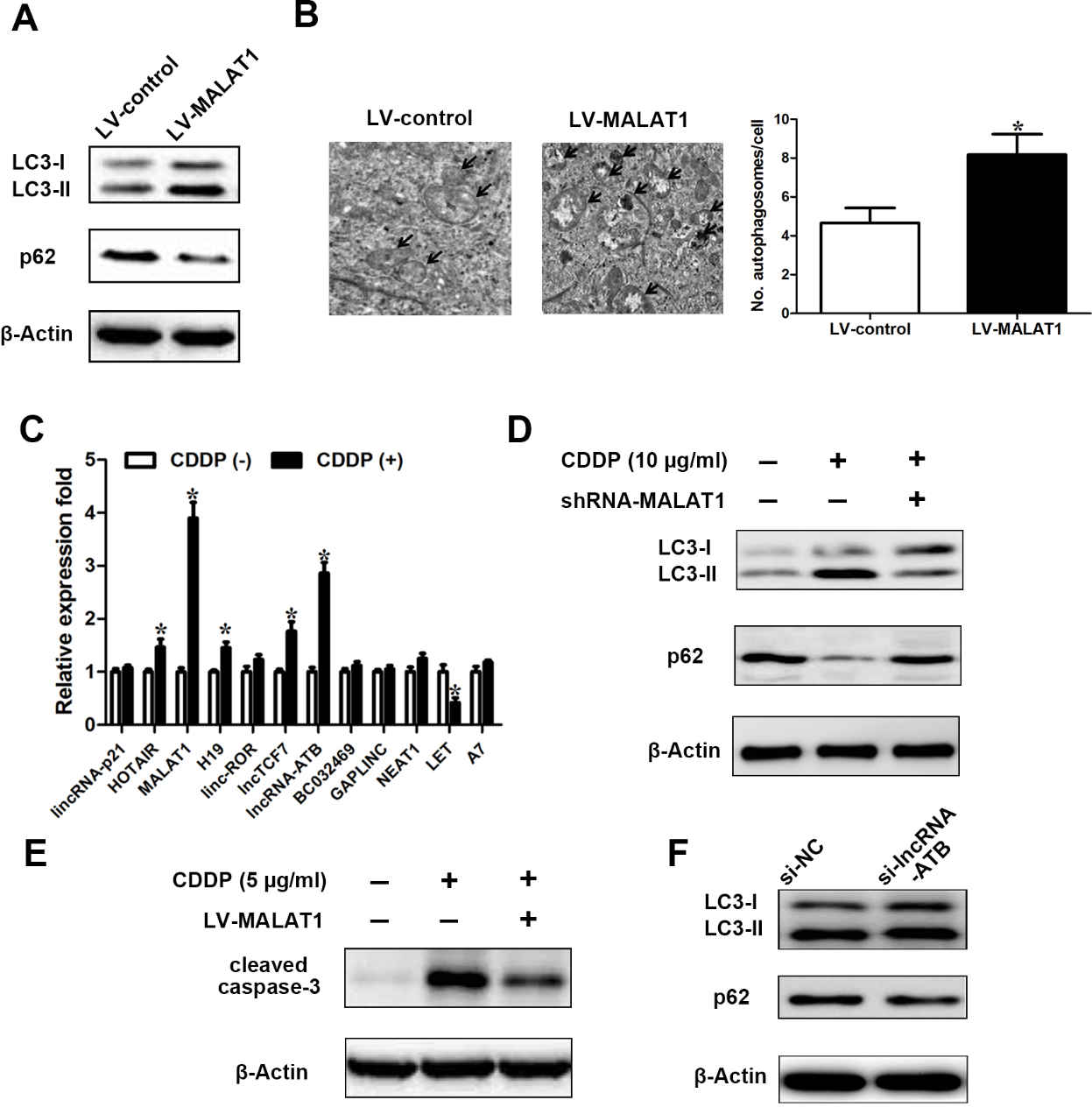


**Figure S4** MALAT1 promotes autophagy. (a) SGC7901/VCR cells stably transfected with full-length human MALAT1 sequence or the empty vector were subjected to Western blot analysis of LC3-II and p62. (b) Autophagy was evaluated using transmission electron microscopy in SGC7901/VCR stably transfected with full-length human MALAT1 sequence or the empty vector. (c) In BGC823 cells, treatment of CDDP (10 µg/ml) for 24 h induced a significant upregulation of MALAT1 as determined with qRT-PCR analysis. (d) In BGC823 cells, treatment of CDDP (10 µg/ml) for 24 h induced a significant activation of autophagy, while MALAT1 knockdown blunted the autophagic response to cisplatin. (e) SGC7901 cells transfected with full-length human MALAT1 sequence or the empty vector were treated with the cisplatin (5 µg/ml) for 24 h, caspase-3 protein was detected by Western blot. The data are presented as the means ± S.D. of values obtained in 3 independent experiments. *, p < 0.05. (f) SGC7901/VCR cells stably transfected with shRNA-lncRNA-ATB or a control were subjected to Western blot analysis of LC3-II and p62.


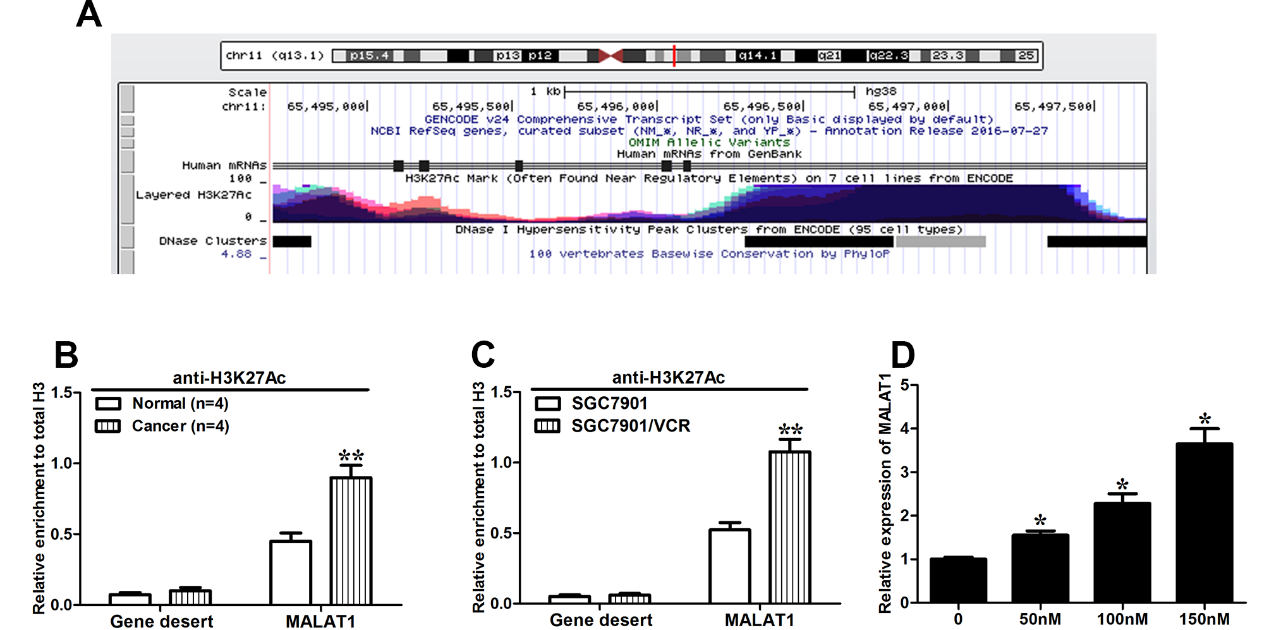


**Figure S5** (a) UCSC Genome Bioinformatics Site (http://genome.ucsc.edu/) showed high enrichment of H3K27Ac at the promoter of MALAT1. (b) ChIP assays detected the H3K27Ac acetylation at promoter of MALAT1 in gastric cancer tissues. (c) ChIP assays detected the H3K27Ac acetylation at promoter of MALAT1 in gastric cancer cells.*, p < 0.05, **, p< 0.01. (d) The expression of the MALAT1 transcript (mean ± standard deviation) was detected using RT-PCR after cells were stimulated with varying concentrations of the histone deacetylase inhibitor trichostatin A (TSA) for 24 hr.


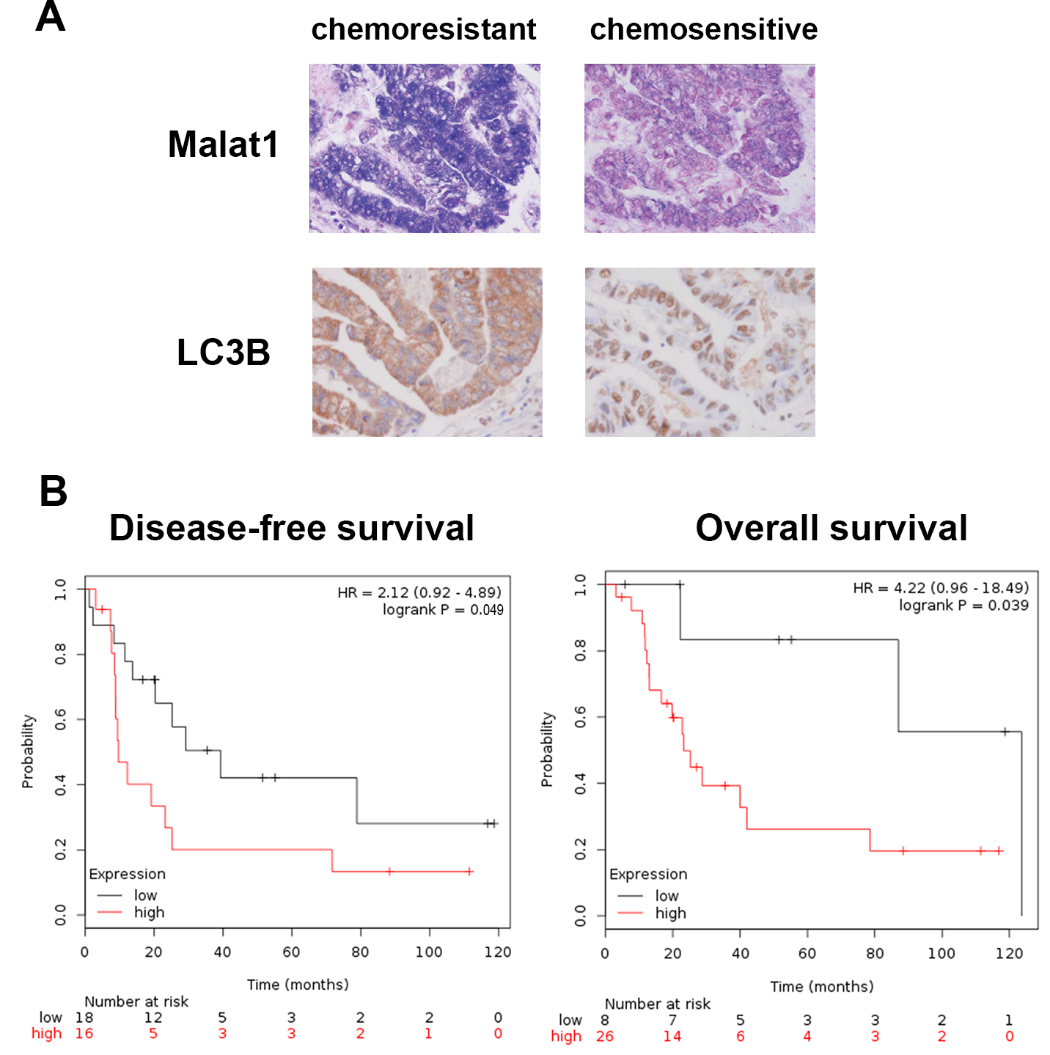


**Figure S6** (a) Compared with chemosensitive patients, LC3B and MALAT1 were markedly upregulated in chemoresistant patients using immunohistochemical analysis (for LC3B) and in situ hybridization analysis (for MALAT1). (b) According to data from The KMPlot database (TCGA), high MALAT1 expression resulted in a poorer disease-free survival (DFS, n=153, p=0.049) and overall survival (OS, n=153, p=0,039) in patients who received 5-Fu based adjuvant therapy

The HRs and p values were calculated with log-rank tests.

**
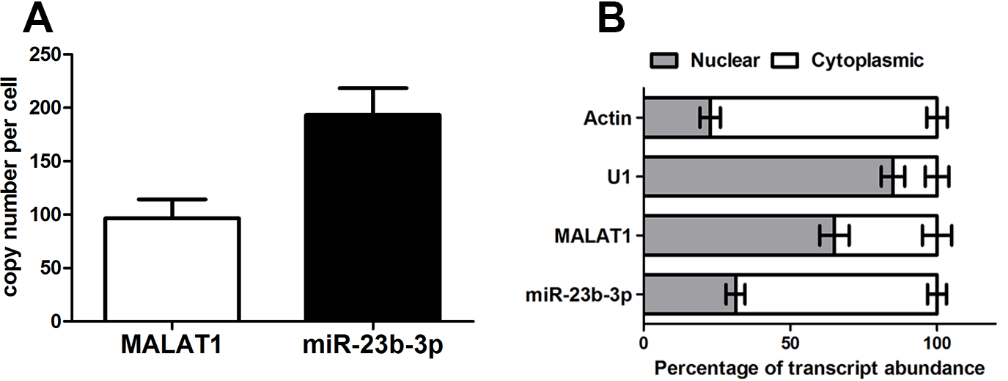
**

**Figure S7** (a) The copy number of MALAT1 or miR-23b-3p detected SGC7901/VCR cells, using RT-PCR and standard curves of known copy numbers of plasmid-derived reference standard. Error bars show standard deviation. (b) Cellular characterization of MALAT1 and miR-23b-3p, the levels of nuclear control transcript (U1), cytoplasmic control transcript (Actin mRNA), and MALAT1 were assessed by qRT-PCR in nuclear and cytoplasmic fractions in SGC7901/VCR cells. Data are presented as a percentage of U1, Actin and MALAT1 levels and total levels for each were taken to be 100%. The data are presented as the means ± S.D. of values obtained in 3 independent experiments.

**
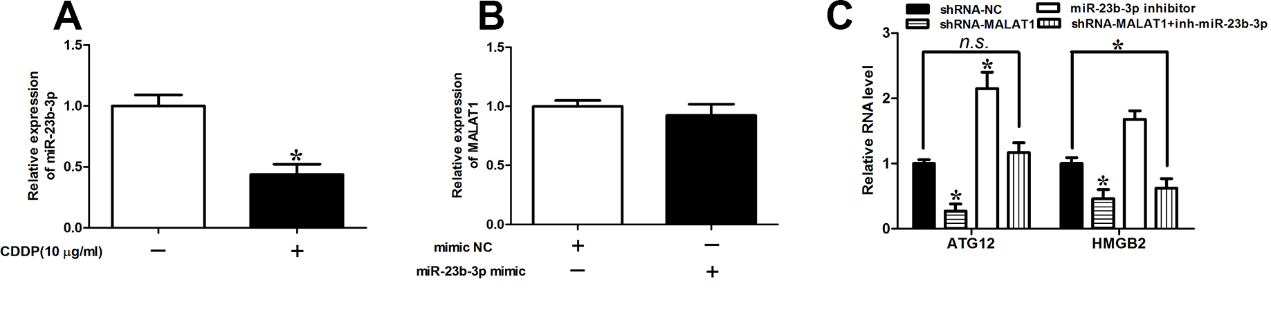
**

**Figure S8** (a) In BGC823 cells, treatment of CDDP (10 µg/ml) for 24 h induced a significant downregulation of miR-23b-3p as determined with qRT-PCR analysis. (b) MALAT1 expression was detected in SGC7901/VCR cells by qRT-PCR after transduction of lentiviruses encoding miR-23b-3p mimic or control mimic. (c) SGC7901/VCR cells were transfected with sh-NC, sh-MALAT1, sh-MALAT1+miRNA-23b-3p inhibitor and miR-23b-3p inhibitor. qRT-PCR was performed 48 h post transfection. ATG12 and HMGB2 were determined with qRT-PCR analysis.

**
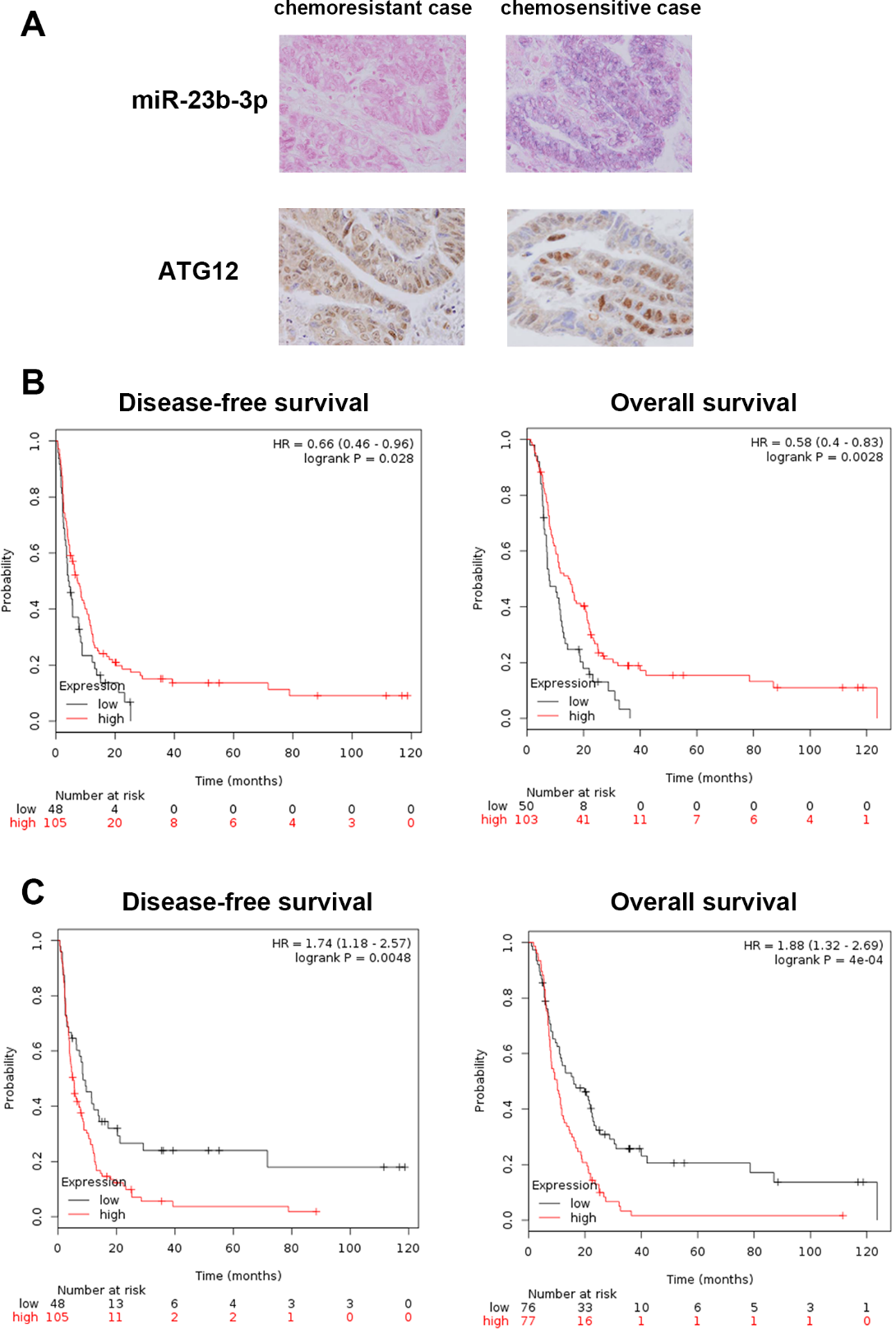
**

**Figure S9** (a) Compared with chemosensitive patients, ATG12 was markedly upregulated and miR-23b-3p was downregulated in chemoresistant patients using immunohistochemical analysis (for ATG12) and in situ hybridization analysis (for miR-23b-3p). (b,c) According to data from The KMPlot database (TCGA), low miR-23b-3p and high ATG12 expression resulted in a poorer disease-free survival and overall survival in patients who received 5-Fu based adjuvant therapy. The HRs and p values were calculated with log-rank tests.

Table S1. Primer sequence used in this study.

| **PCR Primers** |  |
| --- | --- |
| Gene | sequence |
| lincRNA-p21 | 5'- GGGTGGCTCACTCTTCTGGC-3'(forward) |
|  | 5'- TGGCCTTGCCCGGGCTTGTC-3'(reverse) |
| HOTAIR | 5’-CAGTGGGGAACTCTGACTCG-3’(forward) |
|  | 5'-GTGCCTGGTGCTCTCTTACC-3’(reverse) |
| H19 | 5’-ACTCAGGAATCGGCTCTGGAA-3’(forward) |
|  | 5’-CTGCTGTTCCGATGGTGTCTT-3’(reverse) |
| MALAT1 | 5'- AATGTTAAGAGAAGCCCAGGG-3'(forward) |
|  | 5'- AAGGTCAAGAGAAGTGTCAGC-3'(reverse) |
| lncTCF7 | 5’-AGGAGTCCTTGGACCTGAGC-3’(forward) |
|  | 5'-AGTGGCTGGCATATAACCAACA-3’(reverse) |
| LincROR | 5’-TCCCTACTGTTCGTTCACCA-3’(forward) |
|  | 5’-CAGGTTTCCAGATGCGATTT-3’(reverse) |
| LET | 5'-CCTTCCTGACAGCCAGTGTG-3'(forward) |
|  | 5'-CAGAATGGAAATACTGGAGCAAG-3' (reverse) |
| lncRNA-ATB | 5'-TCTGGCTGAGGCTGGTTGAC-3'(forward) |
|  | 5'-ATCTCTGGGTGCTGGTGAAGG-3'(reverse) |
| LncRNA-A7 | 5'CCGTTGGCTCCACAAACCT3' (forward) |
|  | 5'CAGTGACAGTAGCAGGCATCCT3' (reverse) |
| NEAT1 | 5'ACTTGATAACACCCACACCC3'(forward) |
|  | 5'ACAGAGTCACCAGTTTTCCG3'(reverse) |
| miR-23b | 5’-GAGCATCACATTGCCAGGG-3’ (forward) |
|  | 5’-GTCGTATCCAGTGCAGGGTCCGAGGTATTCGCACTGGATACGACGGTAAT-3’ (reverse) |
| ATG12 | 5’-TTTGCTAAAGGCTGTGGG-3’ (forward) |
|  | 5’-AAGGAGCAAAGGACTGAT-3’ (reverse) |
| β-actin | 5’-TCCCTGGAGAAGAGCTACGA-3’(forward) |
|  | 5’-AGCACTGTGTTGGCGTACAG-3’(reverse) |
| GADPH | 5’-GCATCCTGGGCTACACTG-3’(forward) |
|  | 5’-TGGTCGTTGAGGGCAAT-3’(reverse) |
| U6 | 5’-CTCGCTTCGGCAGCACA-3’ (forward) |
|  | 5’-AACGCTTCACGAATTTGCGT-3’ (reverse) |
| Vector construction |  |
| sh-lncRNA-MALAT1-F | 5’-CACCGCTGTGGAGTTCTTAAATATCTTCAAGAGAGATATTTAAGAACTCCACAGCTTTTTTG-3’; |
| sh-lncRNA- MALAT1-R | 5’-GATCCAAAAAAGCTGTGGAGTTCTTAAATATCTCTCTTGAAGATATTTAAGAACTCCACAGC-3’ |
| shRNA-control | 5’-CTCTGCTCTTAAAGATAATTT-3’ |
| PC-F | 5'-GTGAGCTCTGATTCCTGTTCACTACTG-3' |
| PC-R | 5'-GTGTCGACTTATGCCAGGCACCC-3' |
| siRNA-ATG12 | 5'-TTAACCTCTTCCTATCTCA-3' |
| psicheck-MALAT1-F(wt) | 5'-CTGCTTAGTTTGAAAAATGTGACT-3' |
| psicheck-MALAT1-R(wt) | 5'-GGTTAAGTTTTCCAATAATGTGACG-3' |
| psicheck-MALAT1-F(miR-23b-3p) | 5'-CTTTGAAAGACACGATA-3' |
| psicheck-MALAT1-R(miR-23b-3p) | 5'-CTGAGATAGCACCGCTTGC-3' |
| ATG12 3’UTR | 5’-CCTCGAGACCACAAAGAAAATCAACTTGCTAC-3’ (forward) |
|  | 5’-AAAGCGGCCGCACAAACGGTGACTCCCAA-3’ (reverse) |

**Supplemental Materials and Methods**

**Absolute quantification Real time RT-PCR**

We measured the absolute copy number using the standard curve method. The cDNA of MALAT1 was cloned into pcDNA 3.1 vector. The vector was linearized and generated sense RNA transcript using in vitro T7 promoter transcription system (Promega). After digested with RNAse-free DNAse and purification, the transcript was quantified using a spectrophotometer and converted to the number of copies as following formula: Copy number/μl = $\frac{A260\times40\times10-9\times(6.02\times1023)}{(n of A \times329.2) + (n of U \times306.2) + (n of C \times305.2) + (n of G \times345.2)+ 159}$ . The quantified RNA was used as the standard of MALAT1. Synthetic miR-130b-3p RNA was used as the standard of miR-23b-3p. For detection of MALAT1 the standard and total RNA were reverse transcribed with ReverTra Ace (Toyobo) using the reverse primer. The standard of miR-23b-3p were reverse transcribed the same as endogenous miR-23b-3p. The standard cDNA was serially diluted in nuclease-free water. Serial dilutions from 10^6^ to 10^1^ copies were used for standard in a final volume of 20 μl alongside a negative control (RNA) and a non-template control. The SYBR® Premix Ex Taq™ II Kit (Takara) was used for amplification. Quantitative PCR was performed on a CFX96 Real-Time PCR Detection System (Bio-Rad). Absolute quantification determines the actual copy numbers of target genes by relating the Ct value to a standard curve. The data were analyzed by CFX96 software.

**Plasmid construction, lentiviral construction, and cell transfections**

For MALAT1 overexpression, the full-length cDNA of human MALAT1 was synthesized by GeneWiz (Beijing, China) and subcloned to pGC-LV vectors (Genechem Company, Shanghai, China) before sequenced. To produce lentivirus containing MALAT1 gene, HEK-293FT cells were co-transfected with the resulting vector described above, pHelper 1.0 and pHelper 2.0 (Genechem Company, Shanghai, China) using Lipofectamine 2000 according to the manufacturer’s guidelines. Infectious lentiviruses were harvested at 48h post transfection and filtered through 0.45μm PVDF filters, designated LV- MALAT1. For negative control of LV- MALAT1, we used empty vectors containing the green fluorescent protein as the negative control and designated “LV-Control”. Recombinant lentiviruses were concentrated 100-fold by ultracentrifugation (2 h at 50,000 g). The virus-containing pellet was dissolved in DMEM, aliquoted and stored at -80 °C. SGC7901 and SGC7901/VCR cells were infected with concentrated virus at a multiplicity of infection of 80 or 40 in the presence of 8 μg/ml polybrene (Sigma-Aldrich, St. Louis, MO). The supernatant was replaced with complete culture media after 24 h. The expression of MALAT1 in infected cells was confirmed by RT-PCR 96 h after infection.

For stable knockdown of MALAT1, GC cells were transfected with lentiviral constructs encoding MALAT1 shRNA or non-related lncRNA (GenePharma Tech, Shanghai, China). The primer sequence used was as follows: The sequence of shMALAT1 was Sense: 5’-CACCGCTGTGGAGTTCTTAAATATCTTCAAGAGAGATATTTAAGAACTCCACAGCTTTTTTG-3’; Antisense: 5’-GATCCAAAAAAGCTGTGGAGTTCTTAAATATCTCTCTTGAAGATATTTAAGAACTCCACAGC-3’. shRNA-control: 5’-CTCTGCTCTTAAAGATAATTT-3’.

The coding sequence of human ATG12 was amplified and cloned into pcDNA3.1 to generate pcDNA3.1-ATG12.

Cells were grown on six-well plates to 60% confluency, miRNA-23b-3p mimic/negative control mimic 100 nM/ml was transfected into GC cells using Lipofectamine 2000 Reagent (Life Technologies, USA) according to the manufacturer’s instructions.

**Cytosolic/nuclear fractionation**

GC cells (1 × 10^7^) were incubated with hypotonic buffer (25 mM Tris–HCl, PH 7.4, 1 mM MgCl2, 5 mM KCl) on ice for 5 min. An equal volume of hypotonic buffer containing 1% NP-40 was then added, and each sample was left on ice for another 5 min. After centrifugation at 5,000 g for 5 min, the supernatant was collected as the cytosolic fraction. The pellets were re-suspended in nucleus resuspension buffer (20 mM HEPES, pH 7.9, 400 mM NaCl, 1 mM EDTA, 1 mM EGTA, 1 mM DTT, 1 mM PMSF) and incubated at 4°C for 30 min. Nuclear fraction was collected after removing

insoluble membrane debris by centrifugation at 12,000 g for 10 min.

**RNA Pull-Down Assay**

MALAT1 and its antisense RNA were in vitro transcribed from vector pSPT19- MALAT1 and biotin-labeled with the Biotin RNA Labeling Mix (Roche Diagnostics, Indianapolis, IN) and T7/SP6 RNA polymerase (Roche), and purified with an RNeasy Mini Kit (Qiagen, Valencia, CA). One milligram of protein from GC cells extracts was then mixed with 60 pmol of biotinylated RNA, incubated with Dynabeads Myone Streptavidin T1 beads (Invitrogen, Carlbad, CA), and washed. The proteins binding to the streptavidin-coupled dynabeads were resolved by sodium dodecyl sulfate-polyacrylamide gel electrophoresis (SDS-PAGE).
