# Supplementary material for: Long noncoding RNA MALAT1 regulates autophagy associated chemoresistance via miR-23b-3p sequestration in gastric cancer
Source: Mol Cancer. 2017 Nov 21;16:174. doi: 10.1186/s12943-017-0743-3 (PMC5699172; doi:10.1186/s12943-017-0743-3)
Supplement: Supplementary file 2 — Suppemental materials and methods. (DOCX 16 kb) [file 12943_2017_743_MOESM2_ESM.docx]

**Absolute quantification Real time RT-PCR**

We measured the absolute copy number using the standard curve method. The cDNA of MALAT1 was cloned into pcDNA 3.1 vector. The vector was linearized and generated sense RNA transcript using in vitro T7 promoter transcription system (Promega). After digested with RNAse-free DNAse and purification, the transcript was quantified using a spectrophotometer and converted to the number of copies as following formula: Copy number/μl = $\frac{A260\times40\times10-9\times(6.02\times1023)}{(n of A \times329.2) + (n of U \times306.2) + (n of C \times305.2) + (n of G \times345.2)+ 159}$ . The quantified RNA was used as the standard of MALAT1. Synthetic miR-130b-3p RNA was used as the standard of miR-23b-3p. For detection of MALAT1 the standard and total RNA were reverse transcribed with ReverTra Ace (Toyobo) using the reverse primer. The standard of miR-23b-3p were reverse transcribed the same as endogenous miR-23b-3p. The standard cDNA was serially diluted in nuclease-free water. Serial dilutions from 10^6^ to 10^1^ copies were used for standard in a final volume of 20 μl alongside a negative control (RNA) and a non-template control. The SYBR® Premix Ex Taq™ II Kit (Takara) was used for amplification. Quantitative PCR was performed on a CFX96 Real-Time PCR Detection System (Bio-Rad). Absolute quantification determines the actual copy numbers of target genes by relating the Ct value to a standard curve. The data were analyzed by CFX96 software.

**Plasmid construction, lentiviral construction, and cell transfections**

For MALAT1 overexpression, the full-length cDNA of human MALAT1 was synthesized by GeneWiz (Beijing, China) and subcloned to pGC-LV vectors (Genechem Company, Shanghai, China) before sequenced. To produce lentivirus containing MALAT1 gene, HEK-293FT cells were co-transfected with the resulting vector described above, pHelper 1.0 and pHelper 2.0 (Genechem Company, Shanghai, China) using Lipofectamine 2000 according to the manufacturer’s guidelines. Infectious lentiviruses were harvested at 48h post transfection and filtered through 0.45μm PVDF filters, designated LV- MALAT1. For negative control of LV- MALAT1, we used empty vectors containing the green fluorescent protein as the negative control and designated “LV-Control”. Recombinant lentiviruses were concentrated 100-fold by ultracentrifugation (2 h at 50,000 g). The virus-containing pellet was dissolved in DMEM, aliquoted and stored at -80 °C. SGC7901 and SGC7901/VCR cells were infected with concentrated virus at a multiplicity of infection of 80 or 40 in the presence of 8 μg/ml polybrene (Sigma-Aldrich, St. Louis, MO). The supernatant was replaced with complete culture media after 24 h. The expression of MALAT1 in infected cells was confirmed by RT-PCR 96 h after infection.

For stable knockdown of MALAT1, GC cells were transfected with lentiviral constructs encoding MALAT1 shRNA or non-related lncRNA (GenePharma Tech, Shanghai, China). The primer sequence used was as follows: The sequence of shMALAT1 was Sense: 5’-CACCGCTGTGGAGTTCTTAAATATCTTCAAGAGAGATATTTAAGAACTCCACAGCTTTTTTG-3’; Antisense: 5’-GATCCAAAAAAGCTGTGGAGTTCTTAAATATCTCTCTTGAAGATATTTAAGAACTCCACAGC-3’. shRNA-control: 5’-CTCTGCTCTTAAAGATAATTT-3’.

The coding sequence of human ATG12 was amplified and cloned into pcDNA3.1 to generate pcDNA3.1-ATG12.

Cells were grown on six-well plates to 60% confluency, miRNA-23b-3p mimic/negative control mimic 100 nM/ml was transfected into GC cells using Lipofectamine 2000 Reagent (Life Technologies, USA) according to the manufacturer’s instructions.

**Cytosolic/nuclear fractionation**

GC cells (1 × 10^7^) were incubated with hypotonic buffer (25 mM Tris–HCl, PH 7.4, 1 mM MgCl2, 5 mM KCl) on ice for 5 min. An equal volume of hypotonic buffer containing 1% NP-40 was then added, and each sample was left on ice for another 5 min. After centrifugation at 5,000 g for 5 min, the supernatant was collected as the cytosolic fraction. The pellets were re-suspended in nucleus resuspension buffer (20 mM HEPES, pH 7.9, 400 mM NaCl, 1 mM EDTA, 1 mM EGTA, 1 mM DTT, 1 mM PMSF) and incubated at 4°C for 30 min. Nuclear fraction was collected after removing

insoluble membrane debris by centrifugation at 12,000 g for 10 min.

**RNA Pull-Down Assay**

MALAT1 and its antisense RNA were in vitro transcribed from vector pSPT19- MALAT1 and biotin-labeled with the Biotin RNA Labeling Mix (Roche Diagnostics, Indianapolis, IN) and T7/SP6 RNA polymerase (Roche), treated with RNase-free DNase I (Roche) and purified with an RNeasy Mini Kit (Qiagen, Valencia, CA). One milligram of protein from GC cells extracts was then mixed with 60 pmol of biotinylated RNA, incubated with Dynabeads Myone Streptavidin T1 beads (Invitrogen, Carlbad, CA), and washed. The proteins binding to the streptavidin-coupled dynabeads were resolved by sodium dodecyl sulfate-polyacrylamide gel electrophoresis (SDS-PAGE).

.
